# CMR-based planning of catheter and surgical interventions for aortopulmonary collaterals before Fontan completion: pilot study of 5 cases

**DOI:** 10.1186/1532-429X-17-S1-P218

**Published:** 2015-02-03

**Authors:** Keisuke Sato

**Affiliations:** Cardiovascular Center, Mt. Fuji Shizuoka Children’s Hospital, Shizuoka, Japan

## Background

Aortopulmonary collateral arteries (APCAs) are frequently observed in single ventricle patients. However those patients require staged surgery toward Fontan completion, APCAs are believed to cause evil situations against Fontan circulation such as impediment of anterograde pulmonary blood flow, volume overload for single ventricle and decrease in systemic blood flow. Therefore, interventions for APCAs are often considered especially before Fontan completion. Planning of interventions used to be based on fluoroscopic angiogram (FA). But FA can provide the information about the location and size of APCAs. Therefore, FA-based planning depends on the operator's impression about the amount of APCAs. Cardiac MRI (CMR) was reported to useful for quantify APCAs. The aim of this study is to apply CMR to planning of interventions for APCAs before Fontan completion.

## Methods

Five patients with single ventricle before Fontan completion were studied. The diagnosis of five patients was right isomerism for three patients, hypoplastic left heart syndrome for one patient, double inlet of left ventricle for one patient. The patients underwent CMR before and after catheter intervention (coil embolization of APCAs). Velocity-encoded cine phase-contrast sequence was performed for measurement of pulmonary and systemic blood flow. Pulmonary blood flow (Qp) was calculated by total of pulmonary venous flow. Systemic blood flow (Qs) was calculated by total of superior and inferior vena cava flow. APCAs flow was calculated from difference between pulmonary arterial and venous flow. The age of five patients at CMR was 2.58±1.25 years old. Target vessels of coil embolization were decided by each operators on the basis of the information of the amount and location of APCAs.

## Results

APCAs were 21.12±7.22 ml/m^2^ before coil embolization and 17.30±6.25 ml/m^2^ after coil embolization. And the proportion of APCAs to Qp was 55.4±13.9% before coil embolization and 48.1±15.6% after coil embolization. In four cases, the proportion of APCAs to Qp was reduced after coil embolization with CMR-based planning. But the proportion of APCAs to Qp was increased in one case (7%) however it was performed on the basis of CMR planning. Remaining APCAs tended to be located in upper lung, therefore surgical intervention for the branch of subclavian artery was considered.

## Conclusions

CMR-based planning of interventions for APCAs is useful from the aspects of making strategy depending on the amount of APCAs and evaluating the amount of reduced APCAs. But more investigation is needed for the strategy of catheter and surgical interventions for APCAs before Fontan completion with CMR-based planning.

**Figure 1:**
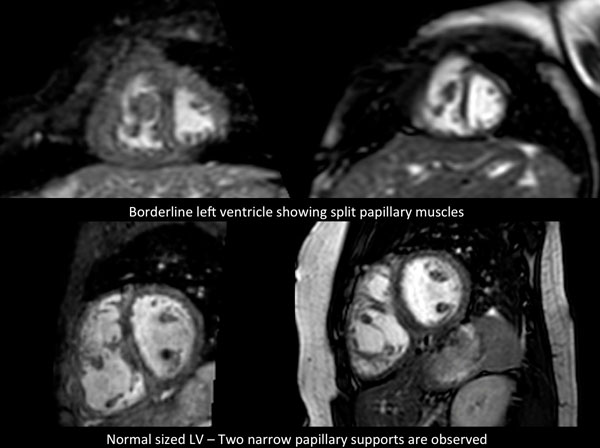
Comparison of normal-sized with borderline LV's.

